# Baseline monocyte count predicts symptom improvement during intravenous ketamine therapy in treatment-resistant depression: a single-arm open-label observational study

**DOI:** 10.3389/fpsyt.2024.1415505

**Published:** 2024-06-24

**Authors:** Bruno Pedraz-Petrozzi, Moritz Spangemacher, Anton Deicher, Lena Drews, Julie Defert, Ana Yaiza Silva-Colmenero, Paul Wein, Elena Riedinger, Gerhard Gründer, Maria Gilles, Alexander Sartorius, Jonathan R. Reinwald

**Affiliations:** ^1^ Department of Psychiatry and Psychotherapy, Central Institute of Mental Health, Medical Faculty Mannheim - University of Heidelberg, Mannheim, Germany; ^2^ Research Group Stress-Related Disorders, Department of Psychiatry and Psychotherapy, Central Institute of Mental Health, Medical Faculty Mannheim - University of Heidelberg, Mannheim, Germany; ^3^ German Centre for Mental Health (Deutsches Zentrum für Psychische Gesundheit, DZPG), Partner Site Heidelberg/Mannheim/Ulm, Mannheim, Germany; ^4^ Department of Molecular Neuroimaging, Central Institute of Mental Health, Medical Faculty Mannheim - University of Heidelberg, Mannheim, Germany; ^5^ Research Group Translational Imaging, Department of Psychiatry and Psychotherapy, Central Institute of Mental Health, Medical Faculty Mannheim - University of Heidelberg, Mannheim, Germany; ^6^ Research Group Systems Neuroscience and Mental Health, Department of Psychiatry and Psychotherapy, University Medical Center Mainz, Johannes Gutenberg University, Mainz, Germany

**Keywords:** ketamine, prediction, treatment response, treatment-resistant depression, inflammation, monocyte, neutrophil, immunity

## Abstract

**Background:**

Neuroinflammatory processes in depression are associated with treatment resistance to conventional antidepressants. Ketamine is an effective new therapeutic option for treatment-resistant depression (TRD). Its well-established immunomodulatory properties are hypothesized to mediate its antidepressant effect. In this context, higher levels of inflammation may predict a better treatment response. However, conclusive evidence for this hypothesis is lacking. We thus investigated whether standard peripheral inflammatory cell markers and C-reactive protein (CRP) levels could predict symptom improvement during intravenous ketamine therapy in TRD patients.

**Methods:**

27 participants with TRD were treated with six weight-adjusted intravenous ketamine infusions (0.5 mg/kg bodyweight) over three weeks. Baseline assessments included CRP, absolute monocyte count (AMC), and absolute neutrophil count (ANC). Depression severity was measured using the Montgomery-Åsberg Depression Rating Scale (MADRS) at baseline (D_1_), after the first (D_3_) and before the last ketamine infusion (D_18_). Raters were blinded for the baseline laboratory assessments.

**Results:**

13 participants responded to ketamine treatment, and 8 participants partially responded. Baseline AMC showed a strong negative correlation with MADRS change at D_3_ (r=-0.57, p=0.002) and at D_18_ (r =-0.48, p=0.010), indicating that a high baseline AMC was associated with greater symptom improvement. A generalized linear model confirmed the association of baseline AMC with symptom improvement during ketamine treatment when additionally accounting for age, sex, and body mass index. Specifically, baseline AMC demonstrated predictive value to discriminate responders and partial responders from non-responders, but lacked discriminative ability between partial responders and responders. Baseline ANC correlated with the MADRS changes at D_3_ (r=-0.39, p=0.046), while CRP values did not correlate at all.

**Conclusions:**

Our prospective single-arm open-label observational study demonstrated that baseline AMC reliably predicted symptom improvement during intravenous ketamine treatment in TRD patients. AMC could therefore serve as a simple and easily accessible marker for symptom improvement during ketamine therapy in daily clinical practice. Future studies with larger sample sizes and a more detailed longitudinal assessment of AMC subtypes are needed to better understand the specific relationship between monocytes and the neuromodulatory effects of ketamine.

## Introduction

Depression is one of the most common and complex diseases worldwide. It affects about 300 million people, representing 4.4% of the global population (1). Treatment-resistant depression (TRD) is a particularly challenging form of the disease that is associated with substantial burden and high economic costs (2). TRD is usually characterized by the failure of at least two pharmacological antidepressant interventions, although definitions vary (3). Different analyses of the STAR*D study data showed markedly heterogeneous results regarding its prevalence, estimating the percentage of depressive patients affected by treatment resistance from 10 to 70% (4–7).

Even though TRD might not reflect a separate neurobiological entity compared to major depressive disorder (MDD), emerging evidence suggests that TRD has distinct neurobiological features (8). One such feature could be elevated inflammation levels (9). Although the influence of the immune system and its role in psychopathology have not yet been sufficiently understood, multiple reviews have reliably demonstrated elevated inflammatory markers in TRD (10, 11). The majority of immune biomarker research has thereby focused on cytokines (12). In this context, some studies suggested that the extent of inflammatory processes could influence or even predict the response to alternative treatment options in TRD (13). The other way round, treatment of TRD might also modulate inflammatory processes and thereby improve psychopathology (14, 15).

One of the most promising treatment approaches for TRD is subanesthetic intravenous infusion therapy with ketamine (16). In various administration forms, accumulating evidence has revealed robust fast-acting antidepressant effects of ketamine and its enantiomer esketamine in patients with TRD (17–19). However, considering that clinical response to ketamine can only be observed in around 50% of TRD patients (20, 21), treatment decisions lack reliable predictors of individual patient benefit. Thus, it would be advantageous to have markers which indicate who will respond to ketamine early in the treatment.

Glutamate levels in the brain are influenced by inflammatory processes (22). Inter alia, ketamine acts as a N-methyl-D-aspartate receptor (NMDA_R_) antagonist, thereby modulating glutamate via the mTOR pathway, so that anti-inflammatory effects have been investigated even before its use in psychiatry (23). Similarly, neuroplasticity, which has been postulated as one of the main mechanisms underlying ketamine’s antidepressant effects (24), seems to have a bidirectional link with inflammation (25). In addition, there is evidence that ketamine has an effect on the gut microbiota, which could be explained by antibacterial and anti-inflammatory processes in the gut-brain axis (26). This raises the question whether the antidepressant effect of ketamine might potentially be mediated by a reduction of inflammatory processes. In this context, higher levels of inflammation could potentially predict a better treatment response in TRD patients. However, evidence for these hypotheses remain inconclusive.

The elevation of the pro-inflammatory cytokine interleukin 6 (IL-6) is one of the most reliable findings in TRD patients (27). Conversely, there have been contradictory results concerning the levels of IL-6 in patients who responded to ketamine. One small study showed a quick decrease of IL-6 after ketamine infusion and that baseline levels of IL-6 predicted antidepressant treatment response (28). This study, however, was criticized for its methodological flaws and could not be replicated in a broader sample (11). Changes in other acute phase proteins, such as C-reactive protein (CRP) or the cytokine tumor necrosis factor-alpha (TNF-α), were also not associated with the response to ketamine (29, 30). However, immune reactions are much more complex, and cytokines reflect only a part of the multi-step processes involved in the immune cascade. Potentially, alterations in the cellular components of the immune system at baseline, which are easily accessible and routinely estimated in patients, could be associated with the therapeutic outcome of ketamine treatment.

Specifically, we hypothesized that higher baseline inflammatory cell counts correlate with symptom improvement during a three-week intravenous ketamine therapy in TRD patients. We focused on absolute monocyte and neutrophil counts at baseline and additionally examined the acute phase protein CRP as a non-cellular marker for acute inflammation. In a next step, we probed whether these baseline inflammatory cell counts provide predictive value for discriminating different therapeutic outcomes during ketamine therapy. Such a marker could be a first step towards a more personalized treatment selection for TRD patients.

## Methods

### Study design

This prospective single-arm open-label observational study was conducted at the Central Institute of Mental Health (CIMH), Mannheim, Germany, from August 2022 to March 2024. It aimed to assess the predictive value of routine clinical inflammatory markers for the treatment response to a three-week-long therapy with intravenous ketamine in patients with TRD. The study was conducted in accordance with ethical principles based on the Declaration of Helsinki and consistent with Good Clinical Practice. The Ethics Committee of the Medical Faculty Mannheim at Heidelberg University approved the protocol (Registration number: 2021–902).

### Participants

All patients received comprehensive information about the purpose and procedure of the study and provided written informed consent to participate in the study. Eligible candidates were screened for inclusion and exclusion criteria and underwent physical and mental examinations. Treatment resistance was required and defined as a lack of clinical response (< 50% improvement in MADRS) to a minimum of two different classes of antidepressants over a period of at least six weeks for each medication in sufficient dosage during the current depressive episode (2). Patients had to be able to prove how long they had been taking the medication and that they had been classified as non-responsive by a healthcare professional during this time. Participants were included in the study if they were at least 18 years old, had a total Montgomery–Åsberg Depression Rating Scale (MADRS) score of ≥ 20 points at the time of the screening, had a current moderate or severe depressive episode according to the 10th version of the International Classification of Diseases (ICD-10), and signed the written informed consent after the examination and verbal explanation of the purposes and procedures of the study. Exclusion criteria comprised bipolar disorder, ketamine treatment during current or past depressive episodes, previous psychotic symptoms, refusal of clinical or laboratory tests or informed consent, and severe medical conditions contraindicating the administration of ketamine. These included, in particular, acute or chronic inflammatory diseases, heart failure, severe arterial hypertension, unstable angina, myocardial or cerebral infarct within the last 12 months, elevated intracranial pressure, severe or treatment-resistant hyperthyroidism, glaucoma, liver cirrhosis or severe hepatic dysfunction, and current (during the last six months before study inclusion) substance use disorder (all except tobacco-related disorders/caffeine-related disorders; alcohol consumption was limited to ≤ 40 gram for men and ≤ 20 gram for women per day, e.g. ≤ two/one large beer (0.5 L) for men/women).

Patients who met all criteria were registered for treatment in the CIMH’s inpatient unit for affective disorders. Here, an experienced psychiatrist re-examined the participant and re-evaluated the criteria for treatment resistant depression and ketamine treatment. Patients were excluded from the study if they decided to discontinue ketamine treatment or withdraw their consent before the last day of treatment. 52 patients were screened for the study and 27 participants were enrolled. All participants completed the study until the last ketamine administration on D_18_.

### Ketamine application

Ketamine was administered intravenously twice a week over a period of three weeks. The first two administrations took place in the inpatient unit for affective disorders. The following four administrations were administered on an outpatient basis in the Early Clinical Trials Unit of the CIMH, if the patient’s condition permitted. Prior to ketamine administration, patients underwent a thorough physical examination including an ECG. No abnormalities in the pre-ketamine examinations were observed. The first ketamine administration was supervised by an anesthetist. No severe side effects were observed. A few patients experienced changes in perception, dissociation, nausea, vomiting, an increase in blood pressure, or headaches, of which none necessitated the intervention of the anesthetist. Dimenhydrinate (i.v.) was administered in cases of nausea or vomiting, and increased blood pressure with hypertensive symptoms (> 160/100 mmHg) was managed with urapidil (i.v.). Ketamine was administered intravenously at a dose of 0.5 mg/kg bodyweight solved in 50 mL of sodium chloride solution using a syringe pump (BRAUN Perfusor - compact plus, Braun Industries, Hesse, Germany) at a rate of 75 mL/h. Each session lasted approximately 45 minutes. All infusions were administered in a low-stimulus environment. Blood pressure and heart rate were monitored regularly during and up to two hours after the ketamine infusion.

### Baseline laboratory assessments

Venous peripheral fasting blood samples were collected at D_1_ between 8 and 10 am for baseline laboratory assessments. For the collection of AMC and ANC, 3 mL EDTA tubes (S-Monovette^®^ EDTA K3E, Sarstedt, Northern Rhine-Westphalia, Germany) and for CRP 7.5 mL serum tubes (Serum-Gel^®^, Sarstedt, Northern Rhine-Westphalia, Germany) were used. After blood sampling, the tubes were transported at 4 degrees Celsius to a clinical laboratory in Mannheim for further analysis. AMC and ANC were determined using the XN 9000/1000 with TS-10/SP-10 Celladivision DI-60, RPU 2100R, and XS-800i (Sysmex Hematology Analyzer, Schleswig-Holstein, Germany). Count procedures were performed automatically for AMC and ANC. For AMC, the laboratory reference ranges varied between 0.19 x 10^9^ and 0.77 x 10^9^ cells/L (men) and 0.29 x 10^9^ and 0.71 x 10^9^ cells/L (women). The laboratory reference ranges for ANC varied between 1.82 x 10^9^ and 7.42 x 10^9^ cells/L (men) and 2.00 x 10^9^ and 7.15 x 10^9^ cells/L (women). Quantitative estimation of CRP was performed using the Cobas c701 analyzer (Roche Industries, Basel, Switzerland) based on an enzymatic particle-enhanced immunological turbidity test. The measuring range was between 0.6 and 350 mg/L, and the dilution limit was 350 mg/L using NaCl 0.9% as a dilution medium with a dilution factor of 2. Reference values were defined as < 5 mg/L. In cases of CRP concentrations under 0.6 mg/L (low detection level), these were presented automatically as < 0.6 after analysis. Regarding the statistical analysis, participants with these findings were included dividing 0.6 by 2, as recommended in the literature (31).

### Psychometric assessments

The Montgomery–Åsberg Depression Rating Scale (MADRS) is an external assessment tool designed as an interview to evaluate psychopathological symptoms of depression. The MADRS is validated in German (32), has a high internal consistency (Cronbach’s alpha = 0.86) (33), and is a sensitive instrument for the changes in psychopathology associated with antidepressant drug treatment (34). Severity grades are typically defined as follows: *mild* (7 to 19 points), *moderate* (20 to 34 points), and *severe* (≥ 35 points) (35). Clinical response was defined by a reduction of the total MADRS scores of ≥50% (36), and remission was defined as a score of 10 or lower after the ketamine treatment (37). The raters of the MADRS were blinded for the results of the baseline laboratory assessments. The Beck Depression Inventory (BDI-II) is a self-report questionnaire for depression, which is also validated in German and has a high internal consistency (Cronbach’s alpha = 0.86) (38). It was used to assess patients’ subjective perception of depressive symptomatology. Higher BDI-II scores represent higher level of depression, with the following classification for depression severity: *mild* (14 to 19 points), *moderate* (20 to 28 points), and *severe* (≥29 points) (33). MADRS and BDI-II were assessed on D_1_, D_3_, and D_18_.

### Statistical analysis

All statistical analysis were performed using the R-based software jamovi 2.5.2 (39) together with the GAMLj toolbox (40). Sample characteristics were described using mean and standard deviation. Shapiro-Wilk test was used to assess normal distribution. All baseline laboratory values were logarithmically transformed due to non-normal distribution. Categorical and count data were presented as numbers or fractions. Bivariate differences between groups were tested using either Fisher’s exact test for categorical variables or Mann-Whitney U-test or Student’s t-test for quantitative variables.

To analyze general differences in depressive symptoms over time, we employed a repeated measures analysis of variance (rmANOVA) for the MADRS and BDI-II scores including D_1_, D_3_, and D_18_. We examined differences in MADRS and BDI-II scores between measurement time points (D_1_, D_3_, and D_18_) and assessed the interaction between response and measurement time points. Effect sizes were quantified using partial eta-square (η²_p_), with interpretations categorized as very small (η²_p_ < 0.01), small (0.01 ≤ η²_p_ < 0.06), moderate (0.06 ≤ η²_p_ < 0.14), and large (η²_p_ ≥ 0.14) (41, 42). When significant differences were detected, Tukey-adjusted *post hoc* comparisons were performed, and a significance threshold of p_tukey_ < 0.05 was defined.

Pearson’s correlation analysis was applied to examine associations between absolute and relative changes in depressive symptoms (MADRS and BDI-II score differences for [D_3_-D_1_] and [D_18_-D_1_]) and baseline clinical laboratory parameters (logCRP, logAMC, and logANC). To estimate an appropriate sample size, a power analysis with G*Power (43) and the appropriate statistical test for a bivariate linear correlation (Pearson’s r) between two non-dichotomous variables (*A priori*: “Correlation: Bivariate Normal Model”) was performed prior to study initiation. Assuming a relatively strong effect with a correlation coefficient of r = 0.5, an α error probability of 0.05, and a power (1-β) of 0.80, we obtained a sample size of 23 participants. Absolute symptom improvement was calculated as the difference between the MADRS scores (e.g. D_3_-D_1_), while relative symptom improvement was calculated as the percentage change compared to the baseline at D_1_ (e.g. ([D_3_-D_1_]/D_1_)*100). P-values were Bonferroni-corrected for multiple comparison (alpha = 0.05, number of investigated parameters = 3, adjusted p-value = 0.0167).

Further, generalized linear models were used to assess the predictive value of baseline logAMC and logANC when accounting for additional factors. Specifically, in addition to logAMC or logANC, age, BMI, and sex were included as independent variables, with absolute and relative changes in MADRS/BDI-II scores serving as the dependent variables. The parameter estimates and their corresponding 95% confidence intervals and p-values were presented in tables. In this context, significance was determined at p < 0.05, considering that the generalized linear models allowed corrections for the abovementioned sample characteristics.

To assess the discriminative ability of logAMC, we plotted receiver operating characteristic (ROC) curves and calculated the area under the ROC curve (AUC) as well as specificity, sensitivity, positive and negative predictive values, and likelihood ratios using the PsychoPDA package (44). The optimal threshold values were determined using Youden’s J statistic (45). For this approach, we used the trichotomized classification of symptom improvement during ketamine treatment at D3 and D18 into non-responders (<25% MADRS improvement), partial responders (25% to 50% MADRS improvement), and responders (>50% MADRS improvement) and compared the three groups with each other. The ROC curves were illustrated with GraphPad Prism version 8.0 (GraphPad Software Inc.; San Diego, CA, USA).

## Results

### Sample characteristics

The sample characteristics are illustrated in **Table 1**. A total of n = 27 patients with TRD (14 females and 13 males) were enrolled in the study. Patients were, on average, 45.44 ± 11.83 years old, had a mean BMI of 27.14 ± 5.94 kg/m^2^, and 6.70 ± 2.30 previous psychiatric medications. The patients’ mean MADRS scores at baseline (28.59 ± 4.34) indicated an overall moderate depression severity, which was confirmed by the BDI-II self-ratings (33.50 ± 11.09). None of the patients had a relevant concomitant physical illness. In particular, there was no evidence of acute or chronic inflammatory diseases. The most common concomitant somatic diseases were arterial hypertension, obstructive sleep aponeurosis syndrome and chronic migraine, in that order. In addition, no patient was taking immunomodulatory medication (e.g., cytostatics, antibiotics, etc.). Baseline ANC was at 0.52 ± 0.14 x 10^9^ cells/L, logAMC at 0.52 ± 0.14 x 10^9^ cells/L, and CRP at 2.33 ± 2.45 mg/dL. One male participant had an AMC value that was above the normal range. This participant was 38 years old, had no previous chronic inflammatory diseases, no infection at the beginning of the study (at the time of blood sampling) and his physical examination was without pathological findings.

**Table 1:** Sample characteristics.

	Mean (SD)
Age of participants	45.44 (11.83)
Sex (female/male)	14/13
BMI (kg/m^2^)	27.14 (5.94)
Response to ketamine i.v. at D_18_ (MADRS reduction > 50%)	13
Partial response to ketamine i.v. at D_18_ (MADRS reduction 25–50%)	8
No response to ketamine i.v. at D_18_ (MADRS reduction <25%)	6
Psychiatric comorbidities (yes/no)	18/9
One comorbidity	14
Two comorbidities	1
Three comorbidities	2
Four comorbidities	1
Somatic comorbidities (yes/no)	19/8
Number of (previous) antidepressant medications	6.70 (2.30)
MADRS scores	
D_1_	28.59 (4.34)
D_3_	18.70 (9.42)
D_18_	15.81 (9.13)
Δ [D_3_-D_1_]	-9.89 (7.89)
% change D_3_ to D_1_	-35.71 (29.14)
Δ [D_18_-D_1_]	-12.78 (8.91)
% changes D_18_ to D_1_	-44.61 (32.57)
BDI-II scores	
D_1_	33.50 (11.09)
D_3_	25.31 (12.84)
D_18_	21.78 (12.80)
Δ [D_3_-D_1_]	-8.76 (8.35)
% changes D_3_ to D_1_	-25.26 (33.31)
Δ [D_18_-D_1_]	-11.85 (8.14)
% changes D_18_ to D_1_	-37.84 (27.71)
Neutrophils (x 10^9^ cells/L)	4.06 (1.51)
Monocytes (x 10^9^ cells/L)	0.52 (0.14)
CRP (mg/dL)	2.33 (2.45)

BDI-II, Beck Depression Inventory; BMI, body mass index; CRP, C-reactive protein; MADRS, Montgomery-Åsberg Depression Rating Scale; D_1_, baseline or day 1; D_3_, day 3; D_18_, day 18; SD, standard deviation.

### Change of depressive symptoms during ketamine treatment

Over the three weeks, 13 TRD patients (4 females, 9 males) responded to the treatment with intravenous ketamine, as defined by a reduction in MADRS of more than 50% at D_18_. 8 participants showed a partial response, as indicated by a 25–50% reduction in MADRS scores. 6 participants (5 females and 1 male) did not respond. We observed no significant differences between the three groups with regard to gender (Fisher’s exact test, p = 0.073), the number of psychiatric comorbidities (Fisher’s exact test, p = 1.000) or previous medications (Spearman’s rho = -0.32, p = 0.106).

**Figure 1:**
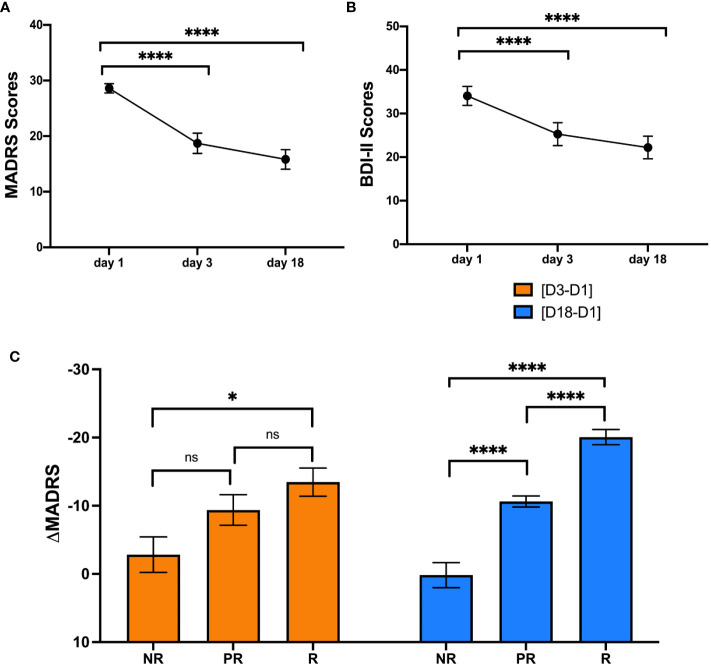
Repeated measures analysis of variance for MADRS and BDI-II scores and comparison of MADRS change between responders, partial responders and non-responders.The MADRS **(A)** and BDI-II scores **(B)** are illustrated as mean ± standard error of the mean at D_1_, D_3_ and D_18_. The bar plots in **(C)** illustrate the change in MADRS score (mean ± standard error of the mean) compared to baseline for D3 (orange, left) and D18 (blue, right) in non-responders, partial responders and responders.BDI-II = Beck Depression Inventory, MADRS = Montgomery-Åsberg Depression Rating Scale, D1 = baseline or day 1, D3 = day 3, D18 = day 18, NR = non-responders, ns = not significant, PR = partial responders, R = responders. * = p < 0.05, **** = p < 0.0001.

Although not the main objective of the study, we could observe a significant decrease of the mean MADRS score over the three-week treatment period (rmANOVA, Mauchly’s W = 0.98, p = 0.766, F_(2, 52)_ = 34.78, p < 0.001, η²_p_ = 0.57; **Figure 1A**). Specifically, the MADRS scores at D_3_ (Tukey-adjusted *post-hoc* comparison, M_Diff_ = 9.89, SE = 1.52, p < 0.0001) and D_18_ (Tukey-adjusted *post-hoc* comparison, M_Diff_ = 12.78, SE = 1.71, p < 0.0001) were significantly lower than on the pre-treatment screening at D_1_. We could further observe a similar decrease in mean BDI-II scores (Mauchly’s W = 1.00, p = 0.999, F_(2, 48)_ = 27.06, p < 0.001, η²_p_ = 0.53; **Figure 1B**) over the treatment period. Again, Tukey-adjusted *post hoc* comparisons indicated significantly lower BDI-II scores at D_3_ (M_Diff_ = 8.76, SE = 1.67, p < 0.0001) and D_18_ (M_Diff_ = 11.84, SE = 1.66, p < 0.0001) in comparison to D_1_. Finally, we could confirm that the differences between responders, partial responders, and non-responders in absolute and relative MADRS scores were significant for [D_3_-D_1_] (Fisher’s one-way ANOVA for absolute MADRS: F_(2,24)_ = 4.87, p = 0.017, Fisher’s one-way ANOVA for relative MADRS: F_(2,24)_ = 4.98, p = 0.016) and for [D_18_-D_1_] (Fisher’s one-way ANOVA for absolute MADRS: F_(2,24)_ = 63.55, p < 0.001, Fisher’s one-way ANOVA for relative MADRS: F_(2,24)_ = 62.70, p < 0.001). *Post-hoc* comparisons showed significant differences between non-responders and responders in absolute and relative MADRS improvement at D3, as well as significant differences between all three groups for absolute and relative MADRS improvement at D18 (**Figure 1C**; **Supplementary Figure S1**). For BDI-II scores, only absolute and relative differences at D18 reached significance (Fisher’s one-way ANOVA for absolute BDI-II: F_(2,23)_ = 3.710, p = 0.040, Fisher’s one-way ANOVA for relative BDI-II: F_(2,23)_ = 4.55, p = 0.022). *Post-hoc* comparisons demonstrated only significant differences between responders and non-responders at D18 for both absolute and relative BDI-II scores (**Supplementary Figures S2, S3**).

### Baseline AMC is associated with the improvement of depressive symptoms during ketamine treatment

Our study primarily aimed to investigate the relationship between peripheral cellular inflammatory markers and CRP at baseline and improvement of depressive symptoms during intravenous ketamine therapy. Thus, we first performed bivariate correlation analyses between changes in depressive symptoms (i.e., [D_3_-D_1_] and [D_18_-D_1_]) and three standard baseline inflammatory markers, namely logANC, logAMC, and logCRP. The results of the correlation analyses are presented in **Table 2**.

**Table 2:** Correlation (Pearson’s r) between change of depressive symptoms and baseline laboratory parameters (logAMC, logANC, and logCRP).

	MADRS		BDI-II	
	[D_3_-D_1_]	[D_18_-D_1_]	[D_3_-D_1_]	[D_18_-D_1_]
**logAMC**	r = -0.57 p = 0.002**	r = -0.48 p = 0.010**	r = -0.20 p = 0.337	r = -0.31 p = 0.123
**logANC**	r = -0.39 p = 0.046	r = -0.32 p = 0.108	r = 0.08 p = 0.720	r = -0.25 p = 0.221
**logCRP**	r = -0.19 p = 0.344	r = -0.19 p = 0.347	r = 0.09 p = 0.666	r = 0.12 p = 0.566

AMC, absolute monocyte count; ANC, absolute neutrophil count; BDI-II, Beck Depression Inventory; CRP, C-reactive protein; D_1_, baseline or day 1; D_3_, day 3; D_18_, day 18; MADRS, Montgomery-Åsberg Depression Rating Scale. All baseline laboratory values were logarithmically transformed due to non-normal distribution. **p < 0.05, Bonferroni corrected for three parameters.

**Figure 2:**
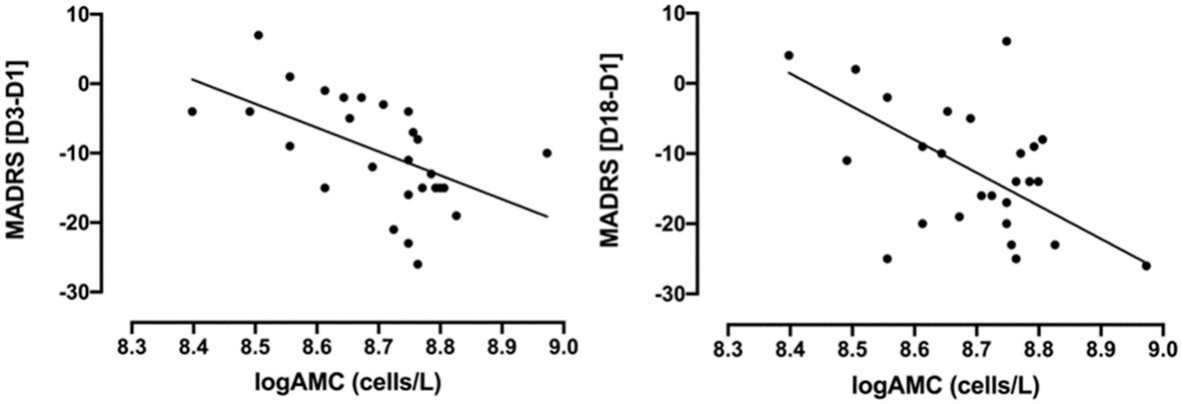
Correlations between absolute changes in MADRS scores (D_3_-D_1_ and D_18_-D_1_) and baseline logAMC (cells/L) in TRD patients treated with intravenous ketamine for three weeks. MADRS [D_3_-D_1_]: Pearson’s r=-0.57, p=0.002; MADRS [D_18_-D_1_]: Pearson’s r =-0.48, p=0.010. AMC, absolute monocyte count; D_1_, baseline or day 1; D_3_, day 3; D_18_, day 18; MADRS, Montgomery-Åsberg Depression Rating Scale; TRD, treatment-resistant depression. AMC was logarithmically transformed due to non-normal distribution.

Most importantly, baseline logAMC negatively correlated to the absolute MADRS change at D_3_ (**Figure 2**, r=-0.57, p=0.002, surviving Bonferroni correction) and at D_18_ (**Figure 2**, r =-0.48, p=0.010, surviving Bonferroni correction). Thus, higher monocytes at baseline were associated with greater treatment response to ketamine, as measured with the MADRS. While baseline logANC correlated with absolute MADRS changes only at D_3_ (r = -0.39, p=0.046, not surviving Bonferroni correction), logCRP did not exhibit any significant association with the absolute MADRS changes. No significant correlations could be observed between the absolute BDI-II changes and the inflammatory markers (**Table 2**), although the correlation with logAMC at D_18_ almost reached trend level.

We further investigated relative MADRS changes, as measured by the relative improvement in relation to the baseline at D_1_, finding similar results (**Supplementary Table S1**). Specifically, logAMC also correlated to the relative MADRS improvement at D_3_ and D_18_, surviving Bonferroni correction at both time points (**Supplementary Figure S4**; **Supplementary Table S1**).

### Baseline AMC predicts symptom improvement during ketamine therapy in a generalized linear model

A generalized linear model (GLM) was performed to evaluate the predictive value of baseline AMC for treatment response when additionally accounting for other factors. In addition to baseline logAMC, age, sex, and BMI were included in the model as explanatory variables. The outcome was quantified using the absolute and relative changes in MADRS scores between D_18_ or D_3_ and D_1_.

The models explained 42% and 34% of the variance in treatment response as measured by the absolute reduction of MADRS scores at D_3_ (MADRS [D_3_-D_1_], R^2^ = 0.42) and D_18_ (MADRS [D_18_-D_1_], R^2^ = 0.34). Baseline logAMC significantly predicted absolute changes of MADRS scores between D_3_ and D_1_ (estimate = -38.43, 95%CI [-59.87; -16.99], p = 0.002; **Table 3**) and between D_18_ and D_1_ (estimate = -31.39, 95%CI [-57.16; -5.63], p = 0.026; **Table 4**). Here, negative beta estimates indicated that higher baseline logAMC were associated with a stronger response to intravenous ketamine treatment. In contrast, no significant effects were observed for age, sex, or BMI (**Tables 3**, **4**). However, baseline logAMC did not predict absolute changes in BDI-II scores between D_3_ and D_1_ (**Supplementary Table S2**) or between D_18_ and D_1_ (**Supplementary Table S3**). Similar results were found for the GLM with the relative MADRS improvement, in which the models explained a variance of 40% and 34%. Here, baseline logAMC also significantly predicted the relative MADRS improvement at D3 and D18 (**Supplementary Tables S4, 5**), but not the relative changes in BDI-II scores at the two time points (**Supplementary Tables S6, 7**).

**Table 3:** Generalized linear model for absolute changes in MADRS score [D_3_-D_1_] in TRD patients treated with intravenous ketamine for three weeks.

Names	95% Confidence Interval					
	Estimate	SE	Lower	Upper	z	p
(Intercept)	-9.91	1.26	-12.37	-7.44	-7.88	< .001
Sex^§^ (f/m)	-1.00	2.75	-6.39	4.39	-0.36	0.719
Age (in years)	-0.17	0.11	-0.39	0.04	-1.58	0.128
BMI (kg/m^2^)	0.25	0.23	-0.19	0.69	1.11	0.277
**logAMC (cells/nL)**	-38.43	10.94	-59.87	-16.99	-3.51	0.002*

AMC, absolute monocyte count; BMI, body mass index; f, female; m, male; SE, standard error of the mean; p, p-value; ^§^reference value was sex = 1 for male. Significant p-values (p < 0.05) are marked with *. AMC was logarithmically transformed due to non-normal distribution.

**Table 4:** Generalized linear model for absolute changes in MADRS score [D_18_-D_1_] in TRD patients treated with intravenous ketamine for three weeks.

Names	95% Confidence Interval					
	Estimate	SE	Lower	Upper	z	P
(Intercept)	-12.82	1.51	-15.78	-9.86	-8.49	< .001
Sex^§^ (f/m)	-2.39	3.30	-8.86	4.09	-0.72	0.478
Age (in years)	-0.08	0.13	-0.34	0.17	-0.63	0.533
BMI (kg/m^2^)	-0.39	0.27	-0.92	0.14	-1.44	0.163
**logAMC**	-31.39	13.14	-57.16	-5.63	-2.39	0.026*

AMC, absolute monocyte count; BMI, body mass index; f, female; m, male; SE, standard error of the mean; p, p-value; ^§^reference value was sex = 1 for male. Significant p-values (p < 0.05) are marked with *. AMC was logarithmically transformed due to non-normal distribution.

We also analyzed the predictive value of baseline ANC for response to intravenous ketamine treatment in TRD patients with a GLM. Again, we included the additional variables age, sex, and BMI and assessed the absolute MADRS improvement at D_3_ and D_18_ (MADRS [D_3_-D_1_], R^2^ = 0.28; MADRS [D_18_-D_1_], R^2^ = 0.29). Baseline logANC was identified as a significant predictor for absolute changes in MADRS scores at D_3_ (MADRS [D_3_-D_1_]: estimate = -22.37, 95%CI [-41.09; -3.66], p = 0.029; **Supplementary Table S8**), but only on a trend-level at D_18_ (MADRS [D_18_-D_1_]: estimate = -20.13, 95%CI [-41.08; 0.82], p = 0.073; **Supplementary Table S9**). In contrast, no significant predictive effects were observed for logANC on relative MADRS improvement at both time points (**Supplementary Tables S10, 11**). Similarly, baseline logANC did neither predict the absolute nor relative change in BDI-II scores at D_3_ (**Supplementary Tables S12, S14**) and D_18_ (**Supplementary Tables S13, S15**).

### Predictive power of baseline AMC is specifically important for distinguishing non-responders from partial responders and responders

**Figure 3:**
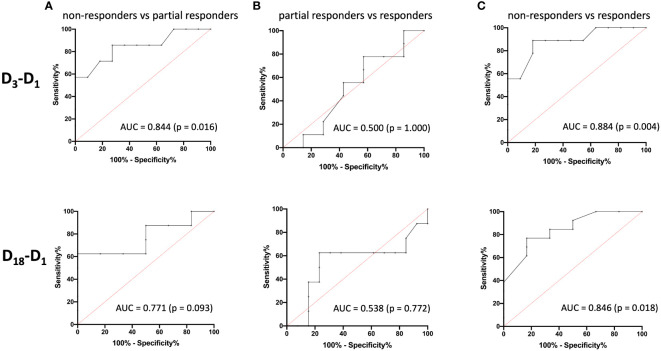
Receiver operating characteristic (ROC) curves and area under the ROC curve (AUC) values for the prediction of treatment response. The ROC curves illustrate the ability of logAMC to discriminate between non-responders and partial responders **(A)**, partial responders and responders **(B)**, and non-responders and responders **(C)** for D_3_ (top) and D_18_ (bottom), respectively. logAMC, log-transformed absolute monocyte count.

To evaluate the predictive performance of baseline AMC, symptom improvement at D_3_ and D_18_ were first divided into the three categories “non-responders” (< 25% MADRS improvement), “partial responders” (25% to 50% MADRS improvement) and “responders” (> 50% MADRS improvement) and then compared with each other using ROC curves (**Figure 3**). The six ROC curves illustrate the trade-off between sensitivity and specificity across different threshold values of log-transformed monocyte concentrations for the comparisons between the three categories at D_3_ and D_18_. Note that the model performed better than random models for the discrimination between non-responders and responders at D_3_ (AUC = 0.884, p = 0.004) and D_18_ (AUC = 0.846, p = 0.018) and between non-responders and partial responders at D_3_ (AUC = 0.844, p = 0.016), while reaching trend-level at D_18_ (AUC = 0.771, p = 0.093). In contrast, the discriminative ability between partial responders and responders was neither significant at D_3_ nor at D_18_. The corresponding sensitivities, specificities, positive and negative predictive values and likelihood-ratios are summarized in **Tables 5**, **6**.

**Table 5:** Sensitivity, specificity, positive predictive value (PPV), negative predictive value (NPV), and likelihood ratios of the MADRS changes D_3_-D_1_ and logAMC.

[D_3_-D_1_]	NR vs. PR	PR vs. R	NR vs. R
Sensitivity (%)	85.71%	42.86%	88.89%
Specificity (%)	72.73%	77.78%	81.82%
PPV (%)	66.67%	60.00%	80.00%
NPV (%)	88.89%	63.64%	90.00%
Youden’s index	0.584	0.206	0.707
Cutpoint (logAMC)	8.69	8.79	8.72
95% CI AUC	[0.644; 1.000]	[0.194; 0.806]	[0.732; 1.000]
LR-	0.196	0.735	0.136
LR+	3.143	1.929	4.889

NR, non-responders; PR, partial responders; R, responders; 95% CI = 95% confidence intervals, LR- = negative likelihood ratio, LR+ = positive likelihood ratio.

**Table 6:** Sensitivity, specificity, positive predictive value (PPV), negative predictive value (NPV), and likelihood ratios of the MADRS changes D_18_-D_1_ and logAMC.

[D_18_-D_1_]	NR vs. PR	PR vs. R	NR vs. R
Sensitivity (%)	62.50%	62.50%	76.92%
Specificity (%)	100%	69.23%	83.33%
PPV (%)	100%	55.56%	90.91%
NPV (%)	66.67%	75.00%	62.50%
Youden’s index	0.625	0.317	0.603
Cutpoint (logAMC)	8.76	8.76	8.71
95% CI AUC	[0.514; 1.000]	[0.254; 0.823]	[0.661; 1.000]
LR-	0.375	0.542	0.277
LR+	Inf	2.031	4.614

NR, non-responders; PR, partial responders; R, responders; 95% CI, 95% confidence intervals; LR-, negative likelihood ratio; LR+, positive likelihood ratio; Inf, infinite number.

## Discussion

Our prospective single-arm open-label observational study investigated whether baseline inflammatory cellular markers or CRP can predict symptom improvement during intravenous ketamine treatment in TRD patients. Most importantly, baseline absolute monocyte count (AMC) was strongly associated with the improvement of depressive symptoms in the MADRS. This effect was already significant 24 hours after the first treatment, suggesting a link to the fast-acting antidepressant properties of ketamine, and remained stable over the three-week treatment period. Further, this association was robust when accounting for other factors, such as age, sex, or BMI, but could not be observed for CRP and only on a trend level for absolute neutrophil count (ANC).

Although our study was not primarily aimed at investigating the treatment effect of intravenous ketamine treatment in TRD patients, we could replicate its well-described antidepressant efficacy (17, 20) as well as its fast-acting properties (46). Specifically, we observed a response to intravenous ketamine treatment after 18 days in approximately 48% of the patients, together with a significant improvement of the MADRS scores already after the first ketamine session. Both numbers are well in line with data from the literature, describing that approximately 50% of TRD patients respond to ketamine treatment (20), with most treatment effects being observed in early stages (47). Thus, despite its relatively small sample size, our study appears to be reasonably representative. In addition, approximately 29% of the patients demonstrated a partial response, as indicated by a MADRS improvement between 25% and 50% at D18.

Importantly, AMC demonstrated the most significant association to the therapeutic outcome of all three inflammatory markers. The correlation coefficients after the first (r = -0.57) and before the last (r = -0.48) ketamine infusion indicate a moderate effect (48), which was stable even when additional factors (age, gender, BMI) were taken into account in the GLM. In summary, TRD patients with a high monocyte count showed a more pronounced improvement of depressive symptoms in the MADRS score during ketamine treatment, while patients with a low AMC showed weaker responses. Baseline AMC could therefore serve as a simple and easily accessible marker for predicting improvement of depressive symptoms during ketamine treatment in clinical practice. Of note, the predictive performance of AMC was particularly effective in distinguishing responders – and to a less strong extent partial responders – from non-responders, but lacked the ability to discriminate between partial responders and responders. Therefore, in everyday daily clinical practice, baseline AMC appear to be more appropriate for determining whether a patient is likely to benefit from ketamine treatment in general, rather than for measuring the extent of the treatment response.

A broad meta-analysis (44 studies (49) suggests that high inflammatory status is associated with non-response to classical antidepressants and thus a hallmark of TRD patients. While most studies have focused on cytokines, some indicate that TRD also leads to changes in immune cells such as monocytes and other macrophages, which are partly responsible for the production of cytokines such as IL-6. Notably, the gene expression of monocyte chemoattractant protein-1 (MCP-1) was significantly increased in white matter samples from depressed suicide completers (50). Kiraly et al. (11) hypothesized that patients under chronic stress experience an increase in chemotactic factors that attracted monocytes to the brain and led to increased mobilization of monocytes from the bone marrow. Specifically, the non-classical fraction of monocytes is proposed to be elevated in chronic inflammatory and autoimmune conditions (51–53). Indeed, patients with MDD showed high levels of the pro-inflammatory cytokines IL-12 and IL-6, increased numbers of non-classical monocytes, and increased activation of classical monocytes in the periphery (54). Further, two studies have demonstrated that pro-inflammatory compounds are associated with an M1-like pro-inflammatory state of monocytes/macrophages (55), and that the presence of ‘inflammatory’ monocytes are correlated with a poor response to antidepressant therapy with serotonergic reuptake inhibitors (56), total sleep deprivation or light therapy (55).

More importantly for our study, Nowak et al. (54) found that a subanesthetic dose of ketamine can significantly reduce the percentage of circulating pro-inflammatory monocytes in mice. Interestingly, the same study showed that subanesthetic ketamine specifically promotes the conversion of monocytes into M2c-like macrophages, thus reducing circulating classical pro-inflammatory monocytes and increasing alternative M2 macrophage subtypes (54). Circulating monocytes and monocytes that traffic to the brain showed increased expression of matrix metalloproteinase 8 (MMP8) both in patients with depression and in chronic stress models in mice (57). These monocytes specifically infiltrated the extracellular space in the CNS and thereby impaired brain function (57). Potentially, via this pathway, ketamine’s effect on monocytes might exert its neuromodulatory properties (**Supplementary Figure S5**).

Branchi et al. (58) proposed that anti-inflammatory treatment might exert a positive effect specifically in people with depression and high baseline inflammatory levels, increasing the efficacy of the treatment on depressive symptoms as well as normalizing immune activation. Ketamine treatment had an immunomodulatory effect via the stimulation of mTOR-associated gene expression receptors, as well as via the programing of human monocytes into M2c-like anti-inflammatory macrophages by inducing high levels of cluster of differentiation 163 (CD163) and Mer tyrosine kinase (MERTK) (54). Such immunomodulatory mechanisms of ketamine potentially explain its positive effect on depressive symptoms especially in patients with higher baseline inflammatory activity and thereby provide a potential neuronal background for the predictive value of the baseline AMC observed in our data.

While we observed a very prominent association between treatment response and baseline AMC, other effects appeared to be more subtle. Only at D_3_, baseline ANC weakly correlated to ketamine treatment response and showed a significant effect in the GLM. Notably, the sample size calculation for our study was designed to detect relatively strong associations, resulting in insufficient power to identify weaker correlations. Indeed, *post-hoc* power analyses (**Supplementary Table S16**) confirmed adequate power for logAMC but indicated insufficient power for detecting effects with logANC and logCRP. Further, the more subtle association of baseline ANC and symptom improvement could possibly be explained by the diverse functions of monocytes and neutrophils during inflammation (59, 60). Chronic inflammatory processes, which are mainly discussed in TRD, usually have a stronger effect on monocyte levels than on neutrophil levels, while the latter tend to reflect predominantly acute inflammatory reactions (61). A more detailed breakdown of the ANC in future studies could help to clarify the specific role of neutrophil subtypes in depression and their potential benefits as a biomarker for ketamine treatment. In line with previous studies, peripheral baseline CRP did not correlate with treatment response (30).

In contrast to the prominent association between AMC and treatment response measured with the MADRS score, the association with changes in the BDI-II scores was not significant. The same applies to ANC. Interestingly, BDI-II scores often change with a temporal delay in comparison to MADRS scores during ketamine treatment (62), suggesting that TRD patients need more time to assess subjective improvement in at least some psychopathological dimensions. This is consistent with our data, in which correlations between BDI-II scores at D_18_ showed a stronger association with AMC compared to D_3_ (r = -0.30 vs. r = -0.17). It is possible that the power of our study was insufficient to unravel such more subtle association.

## Limitations

In general, the small sample size of the study limits the generalizability of the results. However, it is worth noting that the effect size of the current sample, calculated using partial eta squared, and the correlation coefficients suggest a medium-sized to large effect. Our study is further limited by the lack of longitudinal laboratory measurements of inflammation markers during the three-week long treatment period. Thus, we cannot estimate whether the antidepressant effects of ketamine treatment also correlate with a modulation of monocytes or other inflammatory markers during the treatment. This aspect would be crucial to disentangle the immunomodulatory effect of ketamine over time and could provide additional neuromechanistic insights on its action. Discrimination between pro- and anti-inflammatory monocytes has not been performed, and additional inflammatory markers related to monocyte or neutrophil activity, such as the cytokines IL-6 and TNF-α, were neither measured during the ketamine infusion nor at baseline. Finally, no follow-up was performed to evaluate whether the described effects also correlate with a long-lasting antidepressant response.

## Conclusions

Our study proposes baseline AMC as a reliable predictor for response to intravenous ketamine treatment in TRD patients. Its simple accessibility as a routinely examined laboratory marker facilitates seamless integration into daily clinical practices and makes it particularly attractive. In this context, there is an opportunity not only to personalize ketamine as a treatment option, but also to improve the treatment of patients with TRD in general. Nevertheless, further studies are needed to replicate the predictive value of AMC in larger samples and to unravel the neuronal mechanisms underlying the relationship between neuroinflammatory markers in TRD and response to ketamine longitudinally.

## Data availability statement

The datasets presented in this article are not readily available because the datasets generated and analyzed in this study are currently not publicly available but are available from the corresponding author on reasonable request. Requests to access the datasets should be directed to bruno.pedraz@zi-mannheim.de.

## Ethics statement

This study involving humans was approved by the ethics committee of the Medical Faculty Mannheim at Heidelberg University (Registration number: 2021-902). The study was conducted in accordance with the local legislation and institutional requirements. The participants provided their written informed consent to participate in this study.

## Author contributions

BPP: Conceptualization, Data curation, Formal analysis, Investigation, Methodology, Software, Validation, Visualization, Writing – original draft, Writing – review & editing. MS: Conceptualization, Data curation, Investigation, Methodology, Software, Validation, Visualization, Writing – original draft, Writing – review & editing. AD: Data curation, Investigation, Writing – review & editing. LD: Data curation, Investigation, Writing – review & editing. JD: Data curation, Investigation, Writing – review & editing. AYSC: Data curation, Investigation, Writing – review & editing. PW: Data curation, Investigation, Writing – review & editing. ER: Data curation, Investigation, Writing – review & editing. GG: Investigation, Project administration, Resources, Supervision, Writing – review & editing. MG: Conceptualization, Data curation, Investigation, Methodology, Supervision, Writing – review & editing. AS: Conceptualization, Data curation, Funding acquisition, Investigation, Methodology, Project administration, Resources, Supervision, Writing – review & editing. JRR: Conceptualization, Data curation, Formal analysis, Investigation, Methodology, resources, Software, Supervision, Validation, Visualization, Writing – original draft, Writing – review & editing.
