# Supplementary material for: Baseline monocyte count predicts symptom improvement during intravenous ketamine therapy in treatment-resistant depression: a single-arm open-label observational study
Source: Front Psychiatry. 2024 Jun 24;15:1415505. doi: 10.3389/fpsyt.2024.1415505 (PMC11265220; doi:10.3389/fpsyt.2024.1415505)
Supplement: Supplementary file 1 [file DataSheet_1.docx]

**Supplementary material**

**Baseline monocyte count predicts treatment response to intravenous ketamine in treatment-resistant depression**

Pedraz-Petrozzi, B., Spangemacher M., et. al. 2024.





**Figure S1 – Comparison of relative MADRS changes between responders, partial responders and non-responders.**

MADRS = Montgomery-Åsberg Depression Rating Scale, D_1_ = baseline or day 1, D_3_ = day 3, D_18_ = day 18, NR = non-responders, PR = partial responders, R = responders. ns = not significant, p > 0.05, * = p < 0.05, **** = p < 0.0001

**
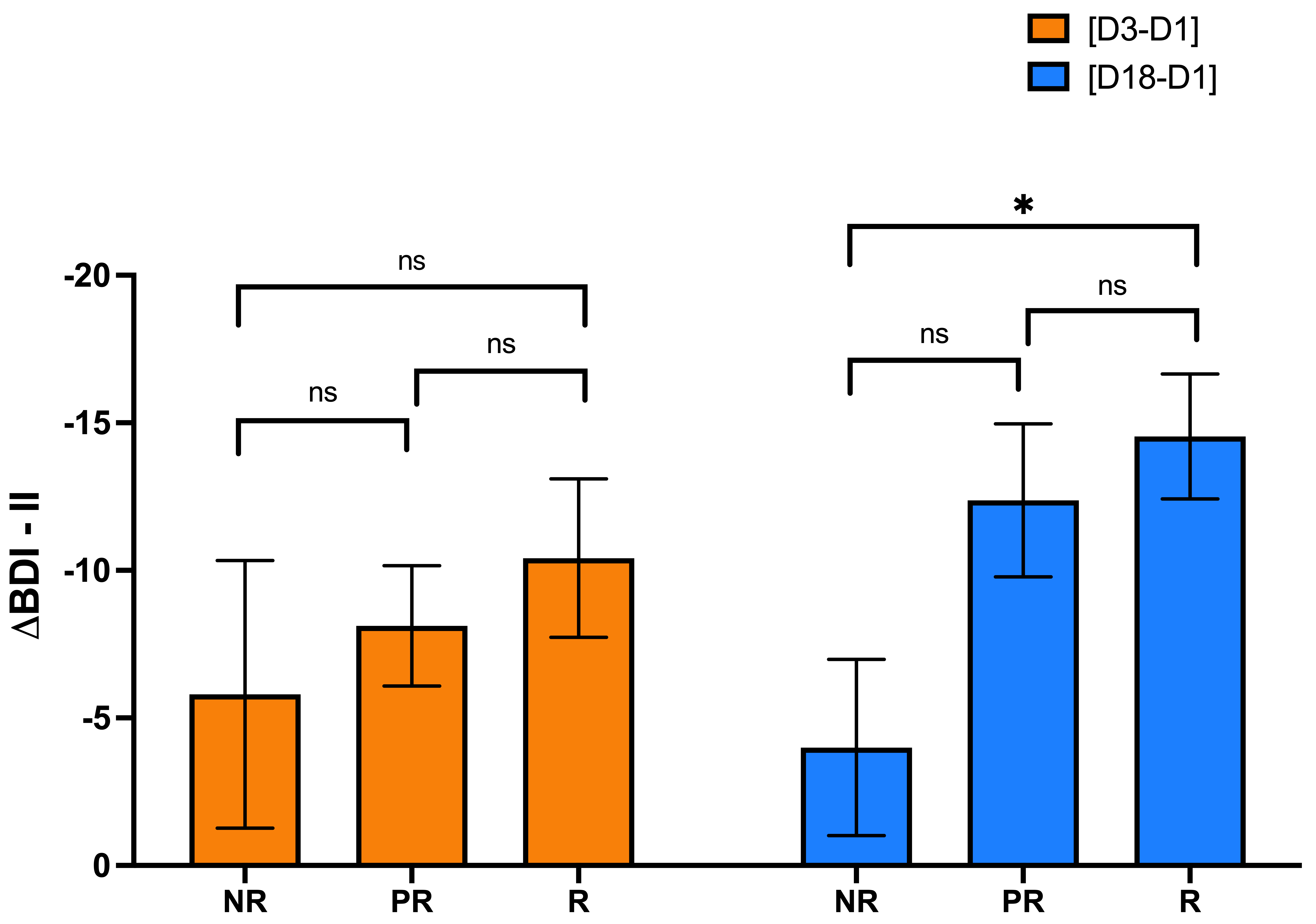
**

**Figure S2 – Comparison of absolute BDI-II change between responders, partial responders and non-responders.**

BDI-II = Beck Depression Inventory, D_1_ = baseline or day 1, D_3_ = day 3, D_18_ = day 18, NR = non-responders, PR = partial responders, R = responders. ns = not significant, p > 0.05; * = p < 0.05.

**
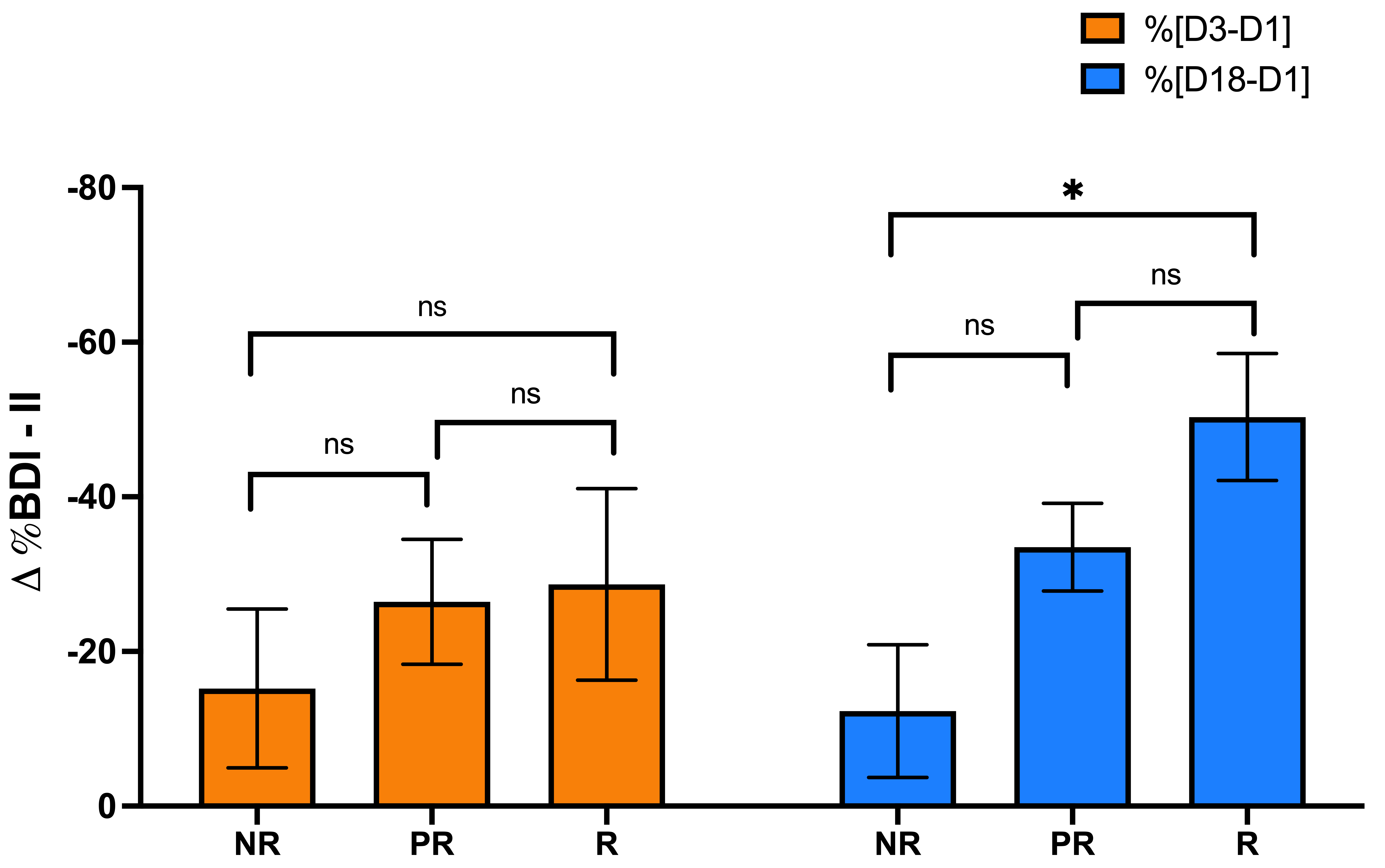
**

**Figure S3 – Comparison of relative BDI-II changes between responders, partial responders and non-responders.**

BDI-II = Beck Depression Inventory, D_1_ = baseline or day 1, D_3_ = day 3, D_18_ = day 18, NR = non-responders, PR = partial responders, R = responders. ns = not significant, p > 0.05,; * = p < 0.05

|  | **MADRS** | | **BDI-II** | |
| --- | --- | --- | --- | --- |
|  | **%[D_3_-D_1_]** | **%[D_18_-D_1_]** | **%[D_3_-D_1_]** | **%[D_18_-D_1_]** |
| **logAMC** | **r = -0.56**  **p = 0.003**** | **r = -0.46**  **p = 0.015**** | r = -0.12  p = 0.557 | r = -0.26  p = 0.206 |
| **logANC** | r = -0.32  p = 0.109 | r = -0.26  p = 0.184 | r = 0.20  p = 0.344 | r = -0.19  p = 0.344 |
| **logCRP** | r = -0.20  p = 0.327 | r = -0.15  p = 0.450 | r = 0.10  p = 0.629 | r = 0.03  p = 0.898 |

**Table S1 - Correlation (Pearson’s r) between relative change of depressive symptoms and baseline laboratory parameters (logAMC, logANC, and logCRP).**

AMC = absolute monocyte count, ANC = absolute neutrophil count, BDI-II = Beck Depression Inventory, CRP = C-reactive protein, D_1_ = baseline or day 1, D_3_ = day 3, D_18_ = day 18, MADRS = Montgomery-Åsberg Depression Rating Scale. All baseline laboratory values were logarithmically transformed due non-normal distribution. ******p < 0.05, Bonferroni corrected for three parameters.


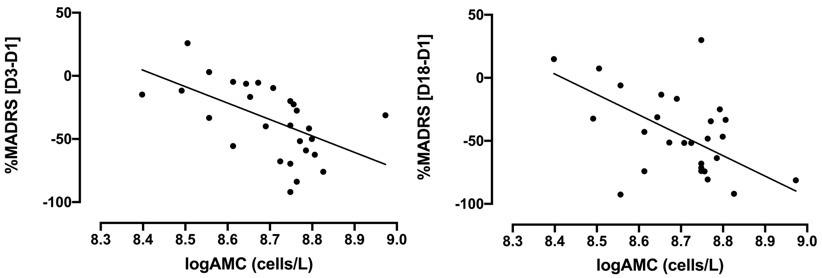


**Figure S4 - Correlations between relative changes in MADRS scores (%D_3_-D_1_ and %D_18_-D_1_) and baseline logAMC in TRD patients treated with intravenous ketamine for three weeks.**

%MADRS [D_3_-D_1_]: Pearson’s r=-0.56, p=0.003; %MADRS [D_18_-D_1_]: Pearson’s r =-0.46, p=0.015

AMC = absolute monocyte count, D_1_ = baseline or day 1, D_3_ = day 3, D_18_ = day 18, MADRS = Montgomery-Åsberg Depression Rating Scale, TRD = treatment-resistant depression. AMC was logarithmically transformed due non-normal distribution

|  | | | | | | | | | | | | | | | |
| --- | --- | --- | --- | --- | --- | --- | --- | --- | --- | --- | --- | --- | --- | --- | --- |
|  | | | | | | | | **95% Confidence Interval** | | | |  | | | |
| **Names** | |  | | **Estimate** | | **SE** | | **Lower** | | **Upper** | | **z** | | **p** | |
| (Intercept) |  |  |  | -8.77 |  | 1.79 |  | -12.27 |  | -5.26 |  | -4.90 |  | < .001 |  |
| Sex^§^ (f/m) |  |  |  | -0.35 |  | 3.87 |  | -7.94 |  | 7.25 |  | -0.09 |  | 0.929 |  |
| Age (in years) |  |  |  | -0.05 |  | 0.15 |  | -0.34 |  | 0.25 |  | -0.31 |  | 0.763 |  |
| BMI (kg/m^2^) |  |  |  | 0.01 |  | 0.32 |  | -0.62 |  | 0.65 |  | 0.04 |  | 0.969 |  |
| logAMC (cells/nL) |  |  |  | -13.32 |  | 15.01 |  | -42.74 |  | 16.11 |  | -0.89 |  | 0.386 |  |

**Table S2 - Generalized linear model for absolute changes in BDI-II score [D_3_-D_1_] in TRD patients treated with intravenous ketamine for three weeks.**

AMC = absolute monocyte count, BMI = body mass index, f = female, m = male, SE = standard error of the mean, p = p-value, ^§^ = reference value was sex = 1 for male. Significant p-values (p < 0.05) are marked with *.

|  | | | | | | | | | | | | | | | | |
| --- | --- | --- | --- | --- | --- | --- | --- | --- | --- | --- | --- | --- | --- | --- | --- | --- |
|  | | | | | | | | **95% Confidence Interval** | | | |  | | | | |
| **Names** | |  | | **Estimate** | | **SE** | | **Lower** | | **Upper** | | **z** | | **p** | | |
| (Intercept) |  |  |  | -11.85 |  | 1.61 |  | -15.00 |  | -8.69 |  | -7.36 |  | < .001 |  |  |
| Sex^§^ (f/m) |  |  |  | -1.12 |  | 3.48 |  | -7.94 |  | 5.70 |  | -0.32 |  | 0.751 |  |  |
| Age (in years) |  |  |  | 0.03 |  | 0.14 |  | -0.24 |  | 0.30 |  | 0.24 |  | 0.812 |  |  |
| BMI (kg/m^2^) |  |  |  | -0.27 |  | 0.29 |  | -0.84 |  | 0.31 |  | -0.91 |  | 0.372 |  |  |
| logAMC (cells/nL) |  |  |  | -17.30 |  | 13.78 |  | -44.31 |  | 9.72 |  | -1.25 |  | 0.223 |  |  |

**Table S3 - Generalized linear model for absolute changes in BDI-II score [D_18_-D_1_] in TRD patients treated with intravenous ketamine for three weeks.**

AMC = absolute monocyte count, BMI = body mass index, f = female, m = male, SE = standard error of the mean, p = p-value, ^§^ = reference value was sex = 1 for male. Significant p-values (p < 0.05) are marked with *.

|  | | | | | | | | | | | | | | | | |
| --- | --- | --- | --- | --- | --- | --- | --- | --- | --- | --- | --- | --- | --- | --- | --- | --- |
|  | | | | | | | | **95% Confidence Interval** | | | |  | | | | |
| **Names** | |  | | **Estimate** | | **SE** | | **Lower** | | **Upper** | | **z** | | **p** | | |
| (Intercept) |  |  |  | -35.87 |  | 4.71 |  | -45.10 |  | -26.64 |  | -7.62 |  | < .001 |  |  |
| Sex^§^ (f/m) |  |  |  | -8.74 |  | 10.31 |  | -28.94 |  | 11.46 |  | -0.85 |  | 0.406 |  |  |
| Age (in years) |  |  |  | -0.59 |  | 0.41 |  | -1.40 |  | 0.21 |  | -1.44 |  | 0.165 |  |  |
| BMI (kg/m^2^) |  |  |  | 0.94 |  | 0.85 |  | -0.72 |  | 2.60 |  | 1.11 |  | 0.277 |  |  |
| **logAMC (cells/nL)** |  |  |  | **-133.32** |  | **40.99** |  | **-213.67** |  | **-52.98** |  | **-3.25** |  | **0.004*** |  |  |

**Table S4 - Generalized linear model for relative changes in MADRS score [D_3_-D_1_] in TRD patients treated with intravenous ketamine for three weeks.**

AMC = absolute monocyte count, BMI = body mass index, f = female, m = male, MADRS = Montgomery-Åsberg Depression Rating Scale, SE = standard error of the mean, p = p-value, ^§^ = reference value was sex = 1 for male. Significant p-values (p < 0.05) are marked with *.

|  | | | | | | | | | | | | | | | | |
| --- | --- | --- | --- | --- | --- | --- | --- | --- | --- | --- | --- | --- | --- | --- | --- | --- |
|  | | | | | | | | **95% Confidence Interval** | | | |  | | | | |
| **Names** | |  | | **Estimate** | | **SE** | | **Lower** | | **Upper** | | **z** | | **p** | | |
| (Intercept) |  |  |  | -44.90 |  | 5.54 |  | -55.75 |  | -34.05 |  | -8.11 |  | < .001 |  |  |
| Sex^§^ (f/m) |  |  |  | -15.98 |  | 12.11 |  | -39.73 |  | 7.76 |  | -1.32 |  | 0.201 |  |  |
| Age (in years) |  |  |  | -0.37 |  | 0.48 |  | -1.31 |  | 0.58 |  | -0.76 |  | 0.457 |  |  |
| BMI (kg/m^2^) |  |  |  | -1.05 |  | 1.00 |  | -3.00 |  | 0.90 |  | -1.06 |  | 0.301 |  |  |
| **logAMC (cells/nL)** |  |  |  | **-102.87** |  | **48.19** |  | **-197.32** |  | **-8.43** |  | **-2.13** |  | **0.044*** |  |  |

**Table S5 - Generalized linear model for relative changes in MADRS score [D_18_-D_1_] in TRD patients treated with intravenous ketamine for three weeks.**

AMC = absolute monocyte count, BMI = body mass index, f = female, m = male, MADRS = Montgomery-Åsberg Depression Rating Scale, SE = standard error of the mean, p = p-value, ^§^ = reference value was sex = 1 for male. Significant p-values (p < 0.05) are marked with *.

|  | | | | | | | | | | | | | | | | |
| --- | --- | --- | --- | --- | --- | --- | --- | --- | --- | --- | --- | --- | --- | --- | --- | --- |
|  | | | | | | | | **95% Confidence Interval** | | | |  | | | | |
| **Names** | |  | | **Estimate** | | **SE** | | **Lower** | | **Upper** | | **z** | | **p** | | |
| (Intercept) |  |  |  | -25.52 |  | 7.12 |  | -39.47 |  | -11.56 |  | -3.58 |  | 0.002 |  |  |
| Sex^§^ (f/m) |  |  |  | -12.66 |  | 15.42 |  | -42.89 |  | 17.56 |  | -0.82 |  | 0.421 |  |  |
| Age (in years) |  |  |  | -0.16 |  | 0.60 |  | -1.34 |  | 1.02 |  | -0.26 |  | 0.794 |  |  |
| BMI (kg/m^2^) |  |  |  | 0.20 |  | 1.28 |  | -2.31 |  | 2.72 |  | 0.16 |  | 0.875 |  |  |
| logAMC (cells/nL) |  |  |  | -21.01 |  | 59.77 |  | -138.15 |  | 96.13 |  | -0.35 |  | 0.729 |  |  |

**Table S6 - Generalized linear model for relative changes in BDI-II score [D_3_-D_1_] in TRD patients treated with intravenous ketamine for three weeks.**

AMC = absolute monocyte count, BMI = body mass index, f = female, m = male, MADRS = Montgomery-Åsberg Depression Rating Scale, SE = standard error of the mean, p = p-value, ^§^ = reference value was sex = 1 for male. Significant p-values (p < 0.05) are marked with *.

|  | | | | | | | | | | | | | | | | |
| --- | --- | --- | --- | --- | --- | --- | --- | --- | --- | --- | --- | --- | --- | --- | --- | --- |
|  | | | | | | | | **95% Confidence Interval** | | | |  | | | | |
| **Names** | |  | | **Estimate** | | **SE** | | **Lower** | | **Upper** | | **z** | | **p** | | |
| (Intercept) |  |  |  | -37.84 |  | 5.42 |  | -48.45 |  | -27.22 |  | -6.99 |  | < .001 |  |  |
| Sex^§^ (f/m) |  |  |  | -10.59 |  | 11.72 |  | -33.55 |  | 12.38 |  | -0.90 |  | 0.377 |  |  |
| Age (in years) |  |  |  | -0.07 |  | 0.47 |  | -0.98 |  | 0.85 |  | -0.14 |  | 0.886 |  |  |
| BMI (kg/m^2^) |  |  |  | -1.03 |  | 0.99 |  | -2.96 |  | 0.90 |  | -1.04 |  | 0.309 |  |  |
| logAMC (cells/nL) |  |  |  | -40.33 |  | 46.38 |  | -131.24 |  | 50.58 |  | -0.87 |  | 0.394 |  |  |

**Table S7 - Generalized linear model for relative changes in BDI-II score [D_18_-D_1_] in TRD patients treated with intravenous ketamine for three weeks.**

AMC = absolute monocyte count, BMI = body mass index, f = female, m = male, MADRS = Montgomery-Åsberg Depression Rating Scale, SE = standard error of the mean, p = p-value, ^§^ = reference value was sex = 1 for male. Significant p-values (p < 0.05) are marked with *.

|  | | | | | | | | | | | | | | | | | |
| --- | --- | --- | --- | --- | --- | --- | --- | --- | --- | --- | --- | --- | --- | --- | --- | --- | --- |
|  | | | | | | | | **95% Confidence Interval** | | | | |  | | | | |
| **Names** | |  | | **Estimate** | | **SE** | | **Lower** | | **Upper** | | **z** | | | **p** | |  |
| (Intercept) |  |  |  | -9.98 |  | 1.40 |  | -12.73 |  | -7.22 |  | -7.10 | |  | < .001 |  |  |
| Sex^§^ (f/m) |  |  |  | -4.73 |  | 3.02 |  | -10.65 |  | 1.19 |  | -1.57 | |  | 0.132 |  |  |
| Age (in years) |  |  |  | -0.13 |  | 0.12 |  | -0.37 |  | 0.12 |  | -1.02 | |  | 0.319 |  |  |
| BMI (kg/m^2^) |  |  |  | 0.31 |  | 0.25 |  | -0.19 |  | 0.81 |  | 1.22 | |  | 0.235 |  |  |
| **logANC (cells/nL)** |  |  |  | **-22.37** |  | **9.55** |  | **-41.09** |  | **-3.66** |  | **-2.34** | |  | **0.029*** |  |  |

**Table S8 - Generalized linear model for absolute changes in MADRS score [D_3_-D_1_] in TRD patients treated with intravenous ketamine for three weeks.**

ANC = absolute neutrophil count, BMI = body mass index, f = female, m = male, SE = standard error of the mean, p = p-value, ^§^ = reference value was sex = 1 for male. Significant p-values (p < 0.05) are marked with *.

|  | | | | | | | | | | | | | | | |
| --- | --- | --- | --- | --- | --- | --- | --- | --- | --- | --- | --- | --- | --- | --- | --- |
|  | | | | | | | | **95% Confidence Interval** | | | |  | | | |
| **Names** | |  | | **Estimate** | | **SE** | | **Lower** | | **Upper** | | **z** | | **p** | |
| (Intercept) |  |  |  | -12.88 |  | 1.57 |  | -15.96 |  | -9.80 |  | -8.19 |  | < .001 |  |
| Sex^§^ (f/m) |  |  |  | -5.53 |  | 3.38 |  | -12.16 |  | 1.09 |  | -1.64 |  | 0.116 |  |
| Age (in years) |  |  |  | -0.04 |  | 0.14 |  | -0.31 |  | 0.23 |  | -0.31 |  | 0.761 |  |
| BMI (kg/m^2^) |  |  |  | -0.34 |  | 0.28 |  | -0.90 |  | 0.22 |  | -1.19 |  | 0.249 |  |
| logANC (cells/nL) |  |  |  | -20.13 |  | 10.69 |  | -41.08 |  | 0.82 |  | -1.88 |  | 0.073 |  |

**Table S9 - Generalized linear model for absolute changes in MADRS score [D_18_-D_1_] in TRD patients treated with intravenous ketamine for three weeks.**

ANC = absolute neutrophil count, BMI = body mass index, f = female, m = male, SE = standard error of the mean, p = p-value, ^§^ = reference value was sex = 1 for male. Significant p-values (p < 0.05) are marked with *.

|  | | | | | | | | | | | | | | | | | |
| --- | --- | --- | --- | --- | --- | --- | --- | --- | --- | --- | --- | --- | --- | --- | --- | --- | --- |
|  | | | | | | | | **95% Confidence Interval** | | | | |  | | | | |
| **Names** | |  | | **Estimate** | | **SE** | | **Lower** | | **Upper** | | **z** | | | **p** | |  |
| (Intercept) |  |  |  | -36.10 |  | 5.28 |  | -46.44 |  | -25.76 |  | -6.84 | |  | < .001 |  |  |
| Sex^§^ (f/m) |  |  |  | -21.31 |  | 11.35 |  | -43.55 |  | 0.93 |  | -1.88 | |  | 0.074 |  |  |
| Age (in years) |  |  |  | -0.43 |  | 0.46 |  | -1.33 |  | 0.47 |  | -0.93 | |  | 0.362 |  |  |
| BMI (kg/m^2^) |  |  |  | 1.13 |  | 0.96 |  | -0.75 |  | 3.00 |  | 1.18 | |  | 0.251 |  |  |
| logANC (cells/nL) |  |  |  | -71.36 |  | 35.87 |  | -141.65 |  | -1.07 |  | -1.99 | |  | 0.059 |  |  |

**Table S10 - Generalized linear model for relative changes in MADRS score [D_3_-D_1_] in TRD patients treated with intravenous ketamine for three weeks.**

ANC = absolute neutrophil count, BMI = body mass index, f = female, m = male, SE = standard error of the mean, p = p-value, ^§^ = reference value was sex = 1 for male. Significant p-values (p < 0.05) are marked with *.

|  | | | | | | | | | | | | | | | |
| --- | --- | --- | --- | --- | --- | --- | --- | --- | --- | --- | --- | --- | --- | --- | --- |
|  | | | | | | | | **95% Confidence Interval** | | | |  | | | |
| **Names** | |  | | **Estimate** | | **SE** | | **Lower** | | **Upper** | | **z** | | **p** | |
| (Intercept) |  |  |  | -45.09 |  | 5.72 |  | -56.30 |  | -33.89 |  | -7.89 |  | < .001 |  |
| Sex^§^ (f/m) |  |  |  | -26.30 |  | 12.30 |  | -50.41 |  | -2.20 |  | -2.14 |  | 0.044 |  |
| Age (in years) |  |  |  | -0.23 |  | 0.50 |  | -1.21 |  | 0.75 |  | -0.46 |  | 0.649 |  |
| BMI (kg/m^2^) |  |  |  | -0.88 |  | 1.04 |  | -2.91 |  | 1.15 |  | -0.85 |  | 0.407 |  |
| logANC (cells/nL) |  |  |  | -66.04 |  | 38.88 |  | -142.23 |  | 10.16 |  | -1.70 |  | 0.103 |  |

**Table S11 - Generalized linear model for relative changes in MADRS score [D_18_-D_1_] in TRD patients treated with intravenous ketamine for three weeks.**

ANC = absolute neutrophil count, BMI = body mass index, f = female, m = male, SE = standard error of the mean, p = p-value, ^§^ = reference value was sex = 1 for male. Significant p-values (p < 0.05) are marked with *.

|  | | | | | | | | | | | | | | | | |
| --- | --- | --- | --- | --- | --- | --- | --- | --- | --- | --- | --- | --- | --- | --- | --- | --- |
|  | | | | | | | | **95% Confidence Interval** | | | |  | | | | |
| **Names** | |  | | **Estimate** | | **SE** | | **Lower** | | **Upper** | | **z** | | **p** | |  |
| (Intercept) |  |  |  | -8.78 |  | 1.82 |  | -12.35 |  | -5.21 |  | -4.83 |  | < .001 |  |  |
| Sex^§^ (f/m) |  |  |  | -0.99 |  | 3.87 |  | -8.58 |  | 6.59 |  | -0.26 |  | 0.800 |  |  |
| Age (in years) |  |  |  | -0.04 |  | 0.15 |  | -0.34 |  | 0.26 |  | -0.25 |  | 0.805 |  |  |
| BMI (kg/m^2^) |  |  |  | -0.02 |  | 0.34 |  | -0.68 |  | 0.64 |  | -0.07 |  | 0.946 |  |  |
| logANC (cells/nL) |  |  |  | 4.21 |  | 12.91 |  | -21.09 |  | 29.52 |  | 0.33 |  | 0.747 |  |  |

**Table S12 - Generalized linear model for absolute changes in BDI-II score [D_3_-D_1_] in TRD patients treated with intravenous ketamine for three weeks.**

ANC = absolute neutrophil count, BMI = body mass index, f = female, m = male, SE = standard error of the mean, p = p-value, ^§^ = reference value was sex = 1 for male. Significant p-values (p < 0.05) are marked with *.

|  | | | | | | | | | | | | | | | | |
| --- | --- | --- | --- | --- | --- | --- | --- | --- | --- | --- | --- | --- | --- | --- | --- | --- |
|  | | | | | | | | **95% Confidence Interval** | | | |  | | | | |
| **Names** | |  | | **Estimate** | | **SE** | | **Lower** | | **Upper** | | **z** | | **p** | |  |
| (Intercept) |  |  |  | -11.85 |  | 1.62 |  | -15.02 |  | -8.68 |  | -7.32 |  | < .001 |  |  |
| Sex^§^ (f/m) |  |  |  | -2.85 |  | 3.42 |  | -9.56 |  | 3.85 |  | -0.83 |  | 0.413 |  |  |
| Age (in years) |  |  |  | 0.05 |  | 0.14 |  | -0.22 |  | 0.33 |  | 0.39 |  | 0.704 |  |  |
| BMI (kg/m^2^) |  |  |  | -0.20 |  | 0.30 |  | -0.79 |  | 0.39 |  | -0.66 |  | 0.515 |  |  |
| logANC (cells/nL) |  |  |  | -13.56 |  | 11.68 |  | -36.44 |  | 9.33 |  | -1.16 |  | 0.259 |  |  |

**Table S13 - Generalized linear model for absolute changes in BDI-II score [D_18_-D_1_] in TRD patients treated with intravenous ketamine for three weeks.**

ANC = absolute neutrophil count, BMI = body mass index, f = female, m = male, SE = standard error of the mean, p = p-value, ^§^ = reference value was sex = 1 for male. Significant p-values (p < 0.05) are marked with *.

|  | | | | | | | | | | | | | | | | |
| --- | --- | --- | --- | --- | --- | --- | --- | --- | --- | --- | --- | --- | --- | --- | --- | --- |
|  | | | | | | | | **95% Confidence Interval** | | | |  | | | | |
| **Names** | |  | | **Estimate** | | **SE** | | **Lower** | | **Upper** | | **z** | | **p** | |  |
| (Intercept) |  |  |  | -25.50 |  | 7.03 |  | -39.27 |  | -11.73 |  | -3.63 |  | 0.002 |  |  |
| Sex^§^ (f/m) |  |  |  | -11.96 |  | 14.94 |  | -41.25 |  | 17.33 |  | -0.80 |  | 0.433 |  |  |
| Age (in years) |  |  |  | -0.17 |  | 0.59 |  | -1.33 |  | 1.00 |  | -0.28 |  | 0.781 |  |  |
| BMI (kg/m^2^) |  |  |  | -0.06 |  | 1.30 |  | -2.62 |  | 2.49 |  | -0.05 |  | 0.961 |  |  |
| logANC (cells/nL) |  |  |  | 41.04 |  | 49.86 |  | -56.69 |  | 138.77 |  | 0.82 |  | 0.420 |  |  |

**Table S14 - Generalized linear model for relative changes in BDI-II score [D_3_-D_1_] in TRD patients treated with intravenous ketamine for three weeks.**

ANC = absolute neutrophil count, BMI = body mass index, f = female, m = male, SE = standard error of the mean, p = p-value, ^§^ = reference value was sex = 1 for male. Significant p-values (p < 0.05) are marked with *.

|  | | | | | | | | | | | | | | | | |
| --- | --- | --- | --- | --- | --- | --- | --- | --- | --- | --- | --- | --- | --- | --- | --- | --- |
|  | | | | | | | | **95% Confidence Interval** | | | |  | | | | |
| **Names** | |  | | **Estimate** | | **SE** | | **Lower** | | **Upper** | | **z** | | **p** | |  |
| (Intercept) |  |  |  | -37.84 |  | 5.41 |  | -48.44 |  | -27.24 |  | -7.00 |  | < .001 |  |  |
| Sex^§^ (f/m) |  |  |  | -14.80 |  | 11.43 |  | -37.21 |  | 7.61 |  | -1.29 |  | 0.210 |  |  |
| Age (in years) |  |  |  | -0.02 |  | 0.46 |  | -0.93 |  | 0.89 |  | -0.04 |  | 0.969 |  |  |
| BMI (kg/m^2^) |  |  |  | -0.85 |  | 1.01 |  | -2.83 |  | 1.13 |  | -0.84 |  | 0.409 |  |  |
| logANC (cells/nL) |  |  |  | -35.35 |  | 39.04 |  | -111.86 |  | 41.17 |  | -0.91 |  | 0.376 |  |  |

**Table S15 - Generalized linear model for relative changes in BDI-II score [D_18_-D_1_] in TRD patients treated with intravenous ketamine for three weeks.**

ANC = absolute neutrophil count, BMI = body mass index, f = female, m = male, SE = standard error of the mean, p = p-value, ^§^ = reference value was sex = 1 for male. Significant p-values (p < 0.05) are marked with *.

|  | **MADRS** | | **BDI-II** | |
| --- | --- | --- | --- | --- |
|  | **[D_3_-D_1_]** | **[D_18_-D_1_]** | **[D_3_-D_1_]** | **[D_18_-D_1_]** |
| **logAMC** | **r = -0.57**  **1-β = 0.945*** | **r = -0.48**  **1-β = 0.836*** | r = -0.20  1-β = 0.262 | r = -0.31  1-β = 0.483 |
| **logANC** | r = -0.39  1-β = 0.662 | r = -0.32  1-β = 0.506 | r = 0.08  1-β = 0.106 | r = -0.25  1-β = 0.355 |
| **logCRP** | r = -0.19  1-β = 0.246 | r = -0.19  1-β = 0.246 | r = 0.09  1-β = 0.115 | r = 0.12  1-β = 0.147 |

**Table S16 – Post-hoc exact test for bivariate correlation models concerning logAMC, logANC, and logCRP. Marked are the power values which overpassed the defined threshold of 0.80.**

AMC = absolute monocyte count, ANC = absolute neutrophil count, CRP = C-reactive protein.


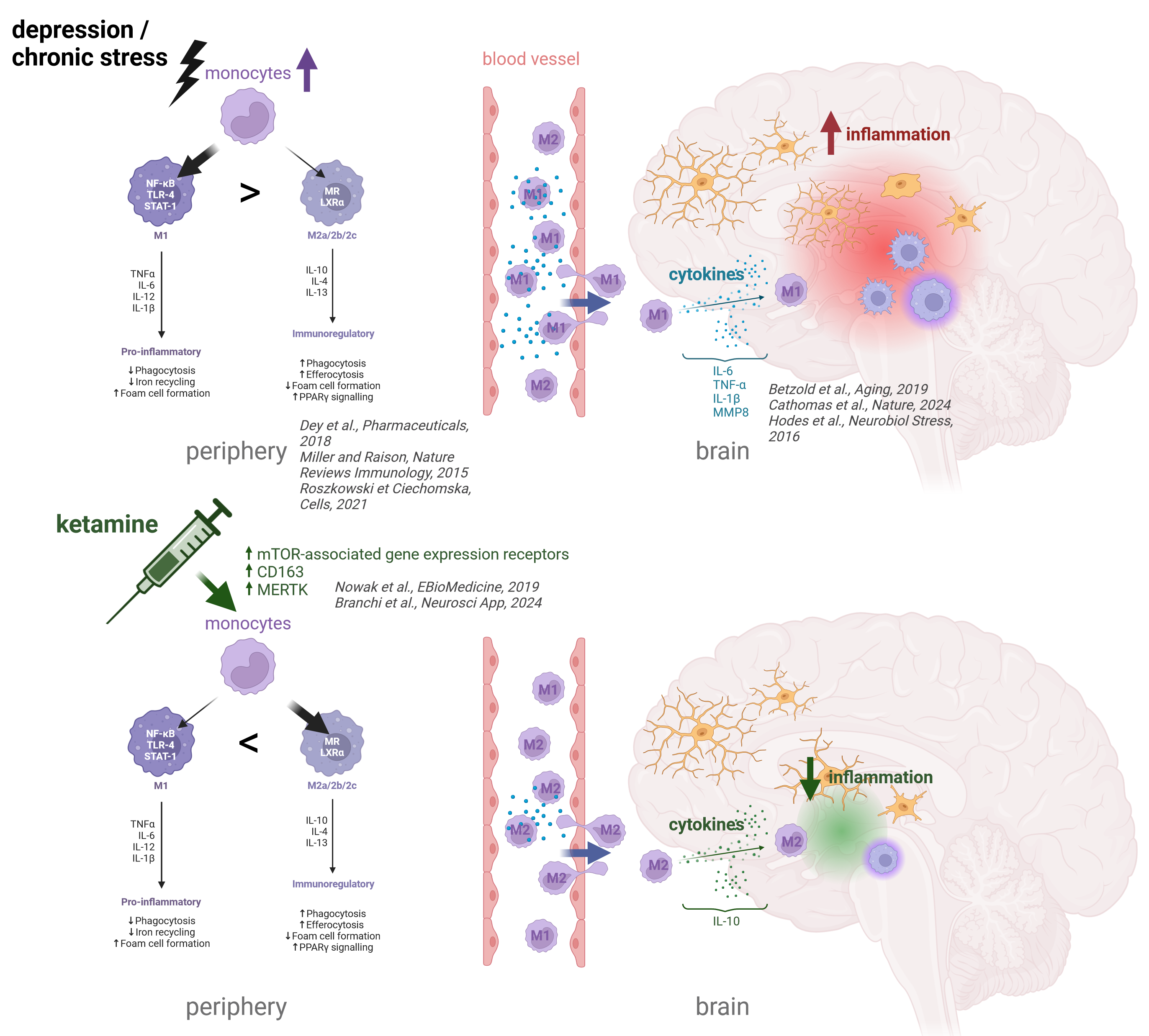


**Figure S5 - Role of monocytes in neuroinflammation and the potential immunomodulatory effects of ketamine on monocytes.** (Created with BioRender.com)
